# Amphiregulin-producing γδ T cells are vital for safeguarding oral barrier immune homeostasis

**DOI:** 10.1073/pnas.1802320115

**Published:** 2018-10-02

**Authors:** Siddharth Krishnan, Ian E. Prise, Kelly Wemyss, Louis P. Schenck, Hayley M. Bridgeman, Flora A. McClure, Tamsin Zangerle-Murray, Conor O’Boyle, Thomas A. Barbera, Faiza Mahmood, Dawn M. E. Bowdish, Dietmar M. W. Zaiss, John R. Grainger, Joanne E. Konkel

**Affiliations:** ^a^The Lydia Becker Institute of Immunology and Inflammation, Faculty of Biology, Medicine and Health, Manchester Academic Health Science Centre, University of Manchester, M13 9PT Manchester, United Kingdom;; ^b^Manchester Collaborative Centre for Inflammation Research, University of Manchester, M13 9NT Manchester, United Kingdom;; ^c^Department of Pathology and Molecular Medicine, McMaster University, Hamilton, ON L8N 3Z5, Canada;; ^d^Institute of Immunology and Infection research, University of Edinburgh, EH9 3FL Edinburgh, United Kingdom

**Keywords:** mucosal immunology, γδ T cells, amphiregulin

## Abstract

γδ T cells are enriched at barrier sites such as the gut, skin, and lung, where their roles in maintaining barrier integrity are well established. However, how these cells contribute to homeostasis at the gingiva, a key oral barrier and site of the common chronic inflammatory disease periodontitis, has not been explored. Here we demonstrate that the gingiva is policed by γδ T cells with a T cell receptor (TCR) repertoire that diversifies during development. Gingival γδ T cells accumulated rapidly after birth in response to barrier damage, and strikingly, their absence resulted in enhanced pathology in murine models of the oral inflammatory disease periodontitis. Alterations in bacterial communities could not account for the increased disease severity seen in γδ T cell-deficient mice. Instead, gingival γδ T cells produced the wound healing associated cytokine amphiregulin, administration of which rescued the elevated oral pathology of *tcrδ*^−/−^ mice. Collectively, our results identify γδ T cells as critical constituents of the immuno-surveillance network that safeguard gingival tissue homeostasis.

## Significance

Loss of oral barrier homeostasis leads to the development of periodontitis, the most common chronic inflammatory condition of mankind. Therefore, it is important to better understand the immune mediators acting at this unique barrier to safeguard tissue integrity. Here we identify a vital role for γδ T cells in constraining pathological inflammation at the oral barrier, as the absence of γδ T cells resulted in enhanced pathology during periodontitis. We show that oral barrier γδ T cells produce the reparative cytokine Amphiregulin, administration of which rescued the elevated oral pathology of *tcrδ*^−/−^ mice. Collectively, we identify a pathway controlling oral immunity mediated by barrier-resident γδ T cells, highlighting that these cells are crucial guards of oral barrier immune homeostasis.

Immunosurveillance networks operating at barrier sites are carefully tailored to each barrier to provide effective immunity. Our understanding of the development and functions of barrier-tailored immune cells has expanded dramatically in recent years, especially when considering the skin, gastrointestinal tract, and lung. In comparison, little is known about the tissue-specific immune mediators safeguarding barrier integrity in the gingiva, the mucosal barrier surrounding the teeth. The gingiva is a unique barrier site, as exposure to commensal and pathogenic microbes occurs concomitantly with high levels of barrier damage arising as a result of mastication (1). Importantly, failure to appropriately control immune responses at the gingiva leads to periodontitis, the most common chronic inflammatory disease of mankind (2). Not only is periodontitis a prevalent disease, but it is also linked to the exacerbation of other inflammatory diseases, including cardiovascular disease and rheumatoid arthritis (3, 4). Moreover, recent advances have highlighted that oral commensal microbes can potentiate diseases at distal sites such as the gut and the joint (5–7). Therefore, delineating the mediators of homeostasis and disease at the gingiva will not only aid development of better therapies for periodontitis but also have broad-reaching implications for systemic inflammation and health.

One key immune population enriched in barrier immunosurveillance networks is γδ T cells. Although these cells remain little explored in the gingiva, studies have implicated γδ T cells in periodontitis pathology. Two studies have shown that gingival γδ T cells produce IL-17A (8, 9); global IL-17A production is a major driver of periodontitis (9, 10). Moreover, IL-17^+^ γδ T cells have been found at sites of inflammation in models of periodontitis (9), and total γδ T cells shown to be elevated in gingival inflammatory infiltrates of patients with periodontitis (11). Thus, as has been shown for other auto-inflammatory diseases, γδ T cells may be drivers of periodontitis pathology.

However, this implied pathogenesis of gingival γδ T cells is at odds with the barrier protective roles of γδ T cells; those resident in other barriers have been shown to safeguard host-commensal interactions (12), ensure epithelial integrity (13), promote barrier repair (14), and coordinate defense against pathogenic challenge (15). Thus, although important functions for γδ T cells in maintaining homeostasis at other barriers are clearly established, how gingiva γδ T cells contribute to oral barrier health and/or disease remains to be explored.

We find that gingiva γδ T cells exhibit distinct phenotypes and functional capabilities compared with γδ T cells policing other barriers at steady-state. We identify a γδ T cell network at the gingiva that is rapidly remodeled after birth to support the unique homeostatic challenges experienced at this site. Importantly, we identify an unrecognized role for γδ T cells in safeguarding gingival homeostasis, as absence of γδ T cells exacerbated periodontitis pathology. Gingival γδ T cells exhibited a unique transcriptional program supporting tissue repair, including production of the epidermal growth factor family member amphiregulin (Areg), administration of which rescued the enhanced periodontitis seen in γδ T cell-deficient mice. Thus, we describe a γδ T cell network vital for limiting oral pathology that is tailored to the gingiva through instruction of Areg production. Our data provide insight into mechanisms ensuring gingival homeostasis that could be exploited to limit periodontitis pathology and the systemic inflammatory consequences of this prevalent disease.

## Results

### Gingival γδ T Cells Are a Heterogeneous Population Maintained by Circulating Precursors.

**Fig. 1.**
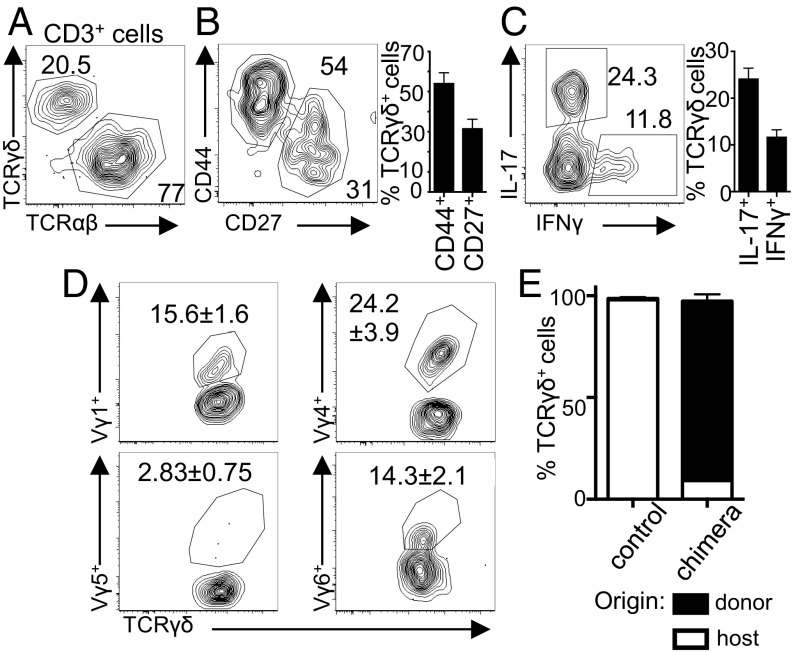
Heterogeneous γδ T cells are resident in the gingiva and can be generated from circulating precursors. (*A*) Representative FACS plot showing TCRβ and TCRγδ staining on live, CD45^+^, CD3^+^ gingiva cells. (*B* and *C*) Representative FACS plot and bar graphs showing percentage γδ T cells positive for (*B*) CD44 and CD27 and (*C*) IL-17 and IFNγ. Data, from five separate experiments. (*D*) Representative FACS plots showing staining of Vγ1, Vγ4, Vγ5, and Vγ6 on adult gingiva γδ T cells. Data representative of four experiments. (*E*) CD45.2^+^ host mice were sublethally irradiated before receipt of CD45.1^+^ donor bone marrow. Bar graph shows degree of chimerism of γδ T cells in gingiva of control and chimeric mice. Data representative of two separate experiments with three to four mice per group. Results are expressed as means ± SEM.

The phenotype and functions of gingival γδ T cells have not been established. We identified a γδ T cell population (Fig. 1*A*), composed of a CD27^+^ and a larger CD44^+^ subset (Fig. 1*B* and *SI Appendix*, Fig. S1*A*). The γδ T cells identified were resident in the gingiva, as evidenced by minimal staining with anti-CD45 after intravenous antibody injection, which distinguishes blood- and tissue-resident cells (*SI Appendix*, Fig. S1*B*). Gingival-resident γδ T cells expressed neither CD4 or CD8α, yet conformed to previously described phenotypes (*SI Appendix*, Fig. S1 *C* and *D*), producing either IFNγ or IL-17 (Fig. 1*C*).

γδ T cells are often classified according to expression of the variable domain of the T cell receptor (TCR)γ (Vγ) chain. The skin, gut, lung, tongue, and urogenital tract are populated by distinctive subsets of γδ T cells bearing tissue-enriched Vγ-chains (16). In contrast to this, the gingiva γδ T cell population did not use a limited TCR repertoire; as determined by flow cytometric staining and nonquantitative PCR, the gingiva was populated by heterogeneous γδ T cells that used Vγ1, Vγ2, Vγ4, and Vγ6 chains (Fig. 1*D* and *SI Appendix*, Fig. S1 *E* and *F*; nomenclature as ref. 17). Thus, unlike other barriers, the gingiva does not contain a restricted γδ T cell population with limited TCR diversity.

As gingival γδ T cells dominantly use Vγ1 and Vγ4, subsets found peripherally and generated in later developmental windows, we queried whether gingival γδ T cells could be replenished from the bone marrow of adult mice. To assess the origin of gingival γδ T cells, we generated bone marrow chimeras, transplanting CD45.1^+^ bone marrow into irradiated CD45.2^+^ recipients. After reconstitution, donor bone marrow was able to regenerate the gingival γδ T cell network (Fig. 1*E* and *SI Appendix*, Fig. S1 *G* and *H*). Combined, our data indicate that gingival γδ T cells are heterogeneous and have the capacity to be replenished by circulating precursors.

### Vγ1^+^ and Vγ4^+^ T Cells Are Rapidly Recruited to the Gingiva After Birth.

**Fig. 2.**
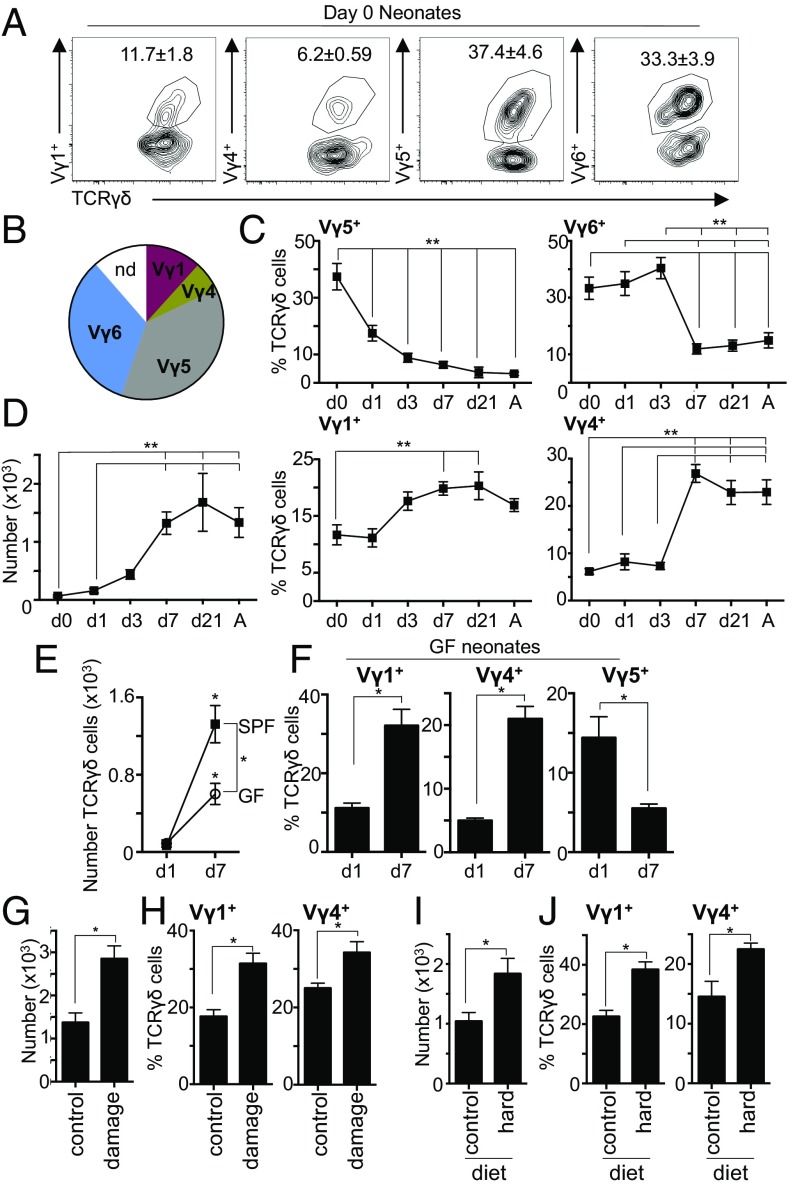
The gingival γδ T cell network is rapidly remodeled after birth. (*A* and *B*) Representative FACS plot and pie chart showing frequencies of Vγ^+^ subsets in the gingiva of day 0 pups (*n* = 6). (*C* and *D*) Graphs showing (*C*) frequencies of specific Vγ^+^ subsets and (*D*) total number of gingival γδ T cells (*A* = adult; *n* = 6–13 mice per time). (*E* and *F*) Graphs show (*E*) total number of gingival γδ T cells and (*F*) frequencies of specific Vγ^+^ subsets in the gingiva of GF mice at day 1 (*n* = 3) and day 7 (*n* = 8) after birth. (*G* and *H*) Graphs show (*G*) total number of gingival γδ T cells and (*H*) frequencies of Vγ^+^ subsets in gingiva of control mice or mice experiencing acute gingival damage. Data representative of two experiments with two to three mice per group. (*I* and *J*) Graphs show (*I*) total number of gingival γδ T cells and (*J*) frequencies of Vγ^+^ subsets in gingiva of mice aged on normal control diet (control) or on a hard pellet diet (hard) since weaning. Data representative of two experiments with two to three mice per group. *P < 0.05 as determined by unpaired Student’s *t* test. **P < 0.05 as determined by one-way ANOVA. Results are expressed as means ± SEM.

In adult gingiva, the γδ T cell population was dominated by subsets predominately found in the periphery. As this is unlike other barriers, we next asked whether during development there was ever a dominant Vγ chain used by gingival γδ T cells. Immediately after birth (day 0), the gingiva γδ T cell network was dominated by cells using Vγ5 and Vγ6 (Fig. 2 *A* and *B*). Although present, Vγ1^+^ and Vγ4^+^ γδ T cells were at much lower frequencies than in adult gingiva, suggesting Vγ5 and Vγ6 subsets initially seed this site. Examining the γδ T cell network in the 1-wk period after birth, we noted it was dramatically remodeled (Fig. 2*C*), occurring at the same time as increases in γδ T cell numbers (Fig. 2*D*). Combined, these data show that, as at other barriers, the gingiva does house specific γδ T cell subsets, with Vγ5 and Vγ6 cells being present at birth. However, rapidly after birth, other Vγ subsets are recruited to the gingiva, and by day 7, they constitute the majority of γδ T cells.

### The Gingival γδ T cell Network Is Remodeled in Response to Barrier Damage, Independent of Commensal Colonization.

We next queried whether gingival bacterial colonization after birth was recruiting Vγ1^+^ and Vγ4^+^ cells and promoting concomitant loss of Vγ5^+^ cells. We examined gingival γδ T cells in germ-free (GF) mice on day 1 and day 7 after birth. Although there was an increase in γδ T cell number after birth, this was reduced compared with conventional, specific-pathogen-free mice (Fig. 2*E*). Despite this reduction in total gingiva γδ T cells, during the neonatal period, GF mice showed increases in Vγ1^+^ and Vγ4^+^ and a loss of Vγ5^+^ cells (Fig. 2*F*), as seen in the specific-pathogen-free mice. As bacterial colonization of the oral cavity occurs during the period being examined, changes to the γδ T cell network in GF mice indicate that microbial signals were not the primary stimulus of this remodeling.

Another stimulus that can shape immunity at the gingiva is mechanical damage (18). At the gingiva, constant barrier damage occurs because of mastication and, during the neonatal period, final tooth development and eruption through the gingiva (19). We queried whether this low-level, chronic damage could promote recruitment of Vγ1^+^ and Vγ4^+^ T cells. As this was difficult to test directly in neonates, we examined whether gingival damage drove recruitment of Vγ1^+^ and Vγ4^+^ cells in adult mice. We addressed this by increasing gingival damage by rubbing the gingiva with a sterile cotton applicator every other day for 10 d, as previously described (18). Acute increases in barrier damage led to increased numbers of gingival γδ T cells and increased frequencies of Vγ1^+^ and Vγ4^+^ T cells (Fig. 2 *G* and *H*).

To further confirm a role for barrier damage in gingival γδ T cell network remodeling, we placed weanling mice on a hardened chow diet, which requires greater mastication forces and causes increased gingival damage (18). As with acute damage, chronic elevation in barrier damage resulted in increases in both total gingival γδ T cells and Vγ1^+^ and Vγ4^+^ subsets (Fig. 2 *I* and *J*). Combined, these data suggest that gingival damage contributes to the gingival γδ T cell network remodeling after birth.

### Gingival Resident γδ T Cells Protect Against Periodontal Bone Loss in Mouse Models of Periodontitis.

**Fig. 3.**
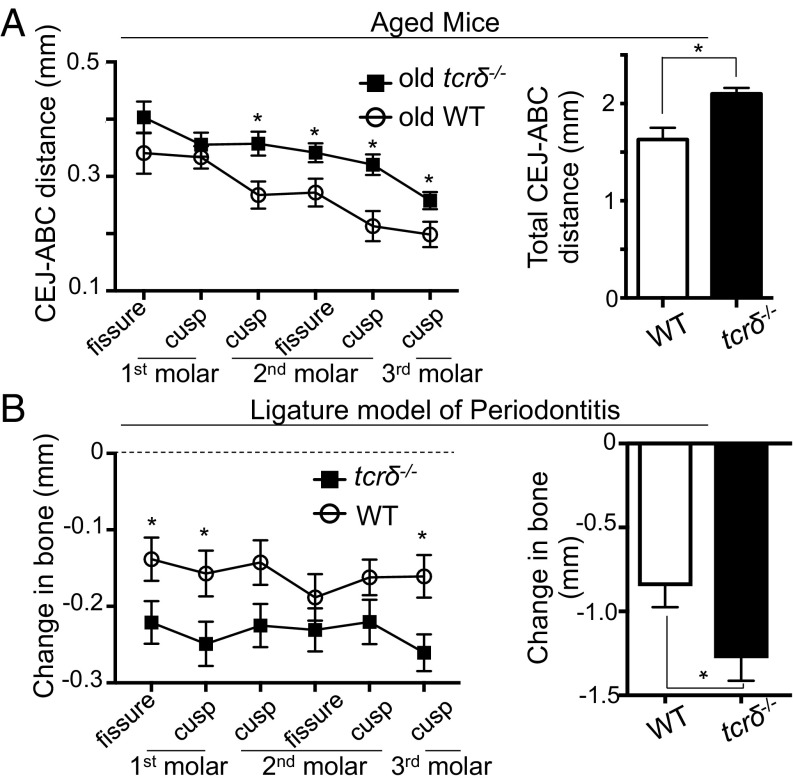
γδ T cells promote gingival homeostasis and protect against periodontal bone loss in mouse models of periodontitis. (*A*) CEJ–ABC distances in maxilla of 24-wk-old wild-type (open circles) and *tcrδ*^−/−^ (closed squares) mice (*n* = 7–12 mice per group). (*Left*) CEJ–ABC distance measured at six defined points across the molars. (*Right*) Graph shows total CEJ–ABC distance. (*B*) CEJ–ABC distances were measured in maxilla of separately housed control and *tcrδ*^−/−^ mice in which experimental periodontitis had been introduced. Change in bone heights was determined by subtracting the CEJ–ABC for periodontitis/ligated molars from naive molars of mice of the same genotype. (*Left*) CEJ–ABC distance measured at six defined points across the molars. (*Right*) Graph shows total change in bone heights in periodontitis mice compared with unligated controls. Data representative of three experiments with four to five mice per group. *P < 0.05 as determined by unpaired Student’s *t* test. Results are expressed as means ± SEM.

We next queried the importance of this unique γδ T cell network, probing the contribution of γδ T cells to gingival homeostasis. Wild-type mice naturally develop periodontal bone loss/periodontitis pathology with age (20), reflecting loss of gingiva-immune homeostasis, which is driven by increased IL-17 (9, 18). As we show gingiva γδ T cells produce IL-17, we hypothesized that periodontitis pathology would be reduced in the absence of γδ T cells. We examined the periodontitis that emerges with age in *tcrδ*^−/−^ mice, which lack γδ T cells. Periodontitis severity is assessed by measuring the distance between the cemento-enamel junction (CEJ) and the alveolar bone crest (ABC); larger distances mean greater bone loss and pathology. We found that in the absence of γδ T cells, periodontal pathology was enhanced (Fig. 3*A*). Aged γδ T cell-deficient mice exhibited increased CEJ–ABC distances, and therefore elevated bone loss compared with controls, suggesting γδ T cells could be promoting homeostasis.

Next we employed an acute model of periodontitis, in which disease is triggered by tissue damage after placement of a ligature around the second molar. This acute gingival injury results in significant periodontal bone loss 10 d after ligature placement. We assessed damage-induced periodontal bone loss in *tcrδ*^−/−^ mice compared with wild-type and found that in the absence of γδ T cells, ligature-induced bone loss was also significantly elevated (Fig. 3*B*). Compared with wild-type, *tcrδ*^−/−^ mice exhibited increased bone loss, as determined by increased changes in bone heights relative to unligated controls.

Further confirming an important role for γδ T cells in limiting periodontitis pathology, treatment of wild-type mice with anti-TCRγδ also resulted in worse pathology in ligature-induced periodontitis (*SI Appendix*, Fig. S2). Combined, our data indicate a critical role for γδ T cells in safeguarding gingival immune homeostasis, as in their absence periodontitis pathology is enhanced.

### Gingival γδ T Cells Control Oral Pathology Independent of the Microbiome.

**Fig. 4.**
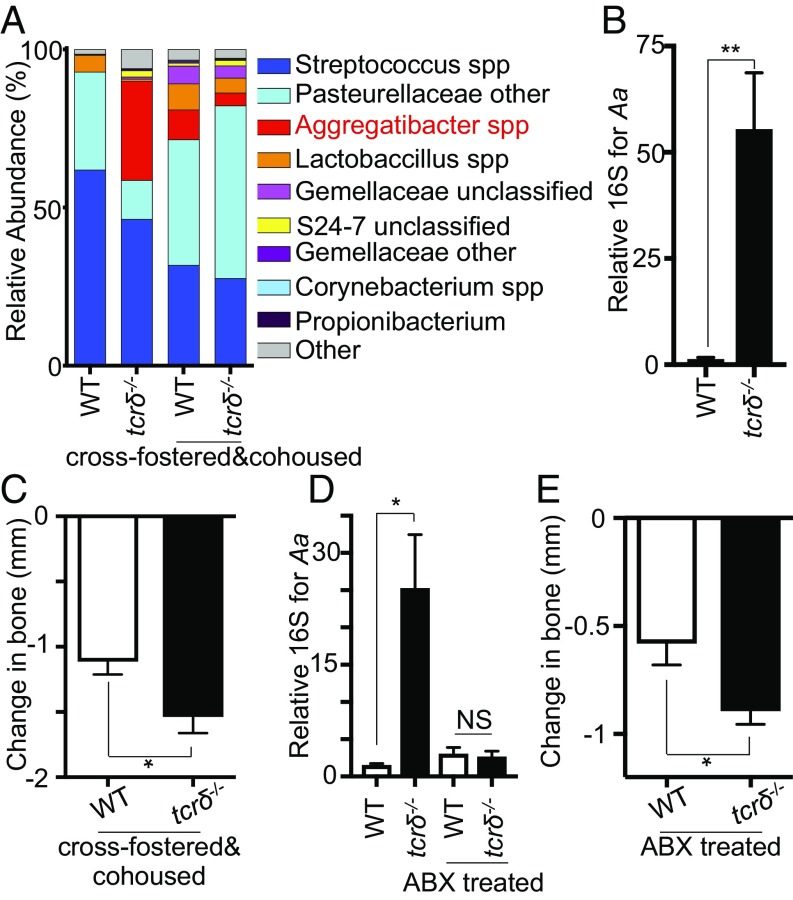
Altered oral microbial communities in the absence of γδ T cells are not responsible for elevated periodontal pathology. (*A*) Graph showing oral microbiome composition depicting most abundant operational taxonomic unit (*n* = 7–10). (*B*) Levels of *Aa* 16S were determined by qPCR assay. Graph shows levels relative to those in control mice. Data representative of two experiments, with four to six mice per group. (*C*) CEJ–ABC distances were measured in maxilla of cross-fostered/cohoused control and *tcrδ*^−/−^ mice in which experimental periodontitis had been introduced. Graph shows total change in bone heights in periodontitis mice compared with unligated control. Data representative of two experiments with three to four mice per group. (*D* and *E*) Experimental periodontitis was induced in control and *tcrδ*^−/−^ mice that had been treated with the antibiotic Sulfamethoxazole-Trimethoprim for 4–5 d before induction and throughout the course of disease. (*D*) Graph shows levels of *Aa* 16S in mice treated with antibiotics, relative to those in control mice, as determined by qPCR. (*E*) CEJ–ABC distances were measured in maxilla of antibiotic-treated control and *tcrδ*^−/−^ mice in which experimental periodontitis had been introduced. Graph shows total change in bone heights in periodontitis mice compared with unligated controls. Data representative of two experiments with four to six mice per group. *P < 0.05, **P < 0.005 as determined by unpaired Students *t* test. Results are expressed as means ± SEM.

Gingival γδ T cells could contribute to the control of local bacteria, and the exacerbation of periodontitis in *tcrδ*^−/−^ mice could be a result of failure to control oral microbes. To define roles for gingival γδ T cells in maintaining this host–microbial dialogue, we examined the oral bacterial community of γδ T cell-deficient mice. Oral bacterial load did not change in *tcrδ*^−/−^ mice (*SI Appendix*, Fig. S3 *A* and *B*). 16S sequencing demonstrated that loss of γδ T cells did not result in any significant bacterial changes at the phyla level, but did result in alterations in oral microbial composition (PERMANOVA *P* < 0.001; *R*^2^ = 0.49; *SI Appendix*, Fig. S3 *C*–*E*). *tcrδ*^−/−^ mice exhibited a significant outgrowth of *Aggregatibacter* species (Fig. 4*A* and *SI Appendix*, Fig. S3*F* and Table S1), suggesting γδ T cells might constrain these microbes. Using PCR approaches, we determined the elevated *Aggregatibacter* spp included *Aggregatibacter actinomycetemcomitans* (*Aa*; Fig. 4*B*), an anaerobic bacteria associated with aggressive periodontitis (21, 22), which could account for the elevated periodontal pathology seen in *tcrδ*^−/−^ mice. To determine whether this was the case, we examined the microbial communities of wild-type and *tcrδ*^−/−^ mice cross-fostered and cohoused from 3 d after birth (cohousing of mice for most of their lifespan). Wild-type mice cross-fostered with *tcrδ*^−/−^ mice contained *Aggregatibacter spp* in their oral microbial communities, although at lower levels than single-housed *tcrδ*^−/−^ animals (Fig. 4*A* and *SI Appendix*, Fig. S3*F*). To ascertain whether elevated levels of *Aa* were contributing to the increased periodontitis pathology seen in *tcrδ*^−/−^ mice, we performed ligature-induced periodontitis on cross-fostered and cohoused wild-type and *tcrδ*^−/−^ mice. Despite similar abundances of oral *Aggregatibacter spp*, *tcrδ*^−/−^ mice still exhibited elevated periodontal pathology compared with cross-fostered and cohoused wild-type controls (Fig. 4*C* and *SI Appendix*, Fig. S3*G*).

Next, we treated separately housed wild-type and *tcrδ*^−/−^ mice with antibiotics to deplete levels of oral *Aa* (Fig. 4*D*). Even when oral *Aa* was substantially reduced, *tcrδ*^−/−^ mice still exhibited elevated periodontitis pathology compared with controls (Fig. 4*E* and *SI Appendix*, Fig. S3*H*). These data demonstrate that outgrowth of *Aa* in *tcrδ*^−/−^ mice cannot alone account for the elevated oral pathology seen in these mice, indicating that gingival γδ T cells exhibit additional functional properties to limit periodontitis pathology.

### Gingival γδ T Cells Produce the Cytokine Areg to Limit Oral Pathology.

**Fig. 5.**
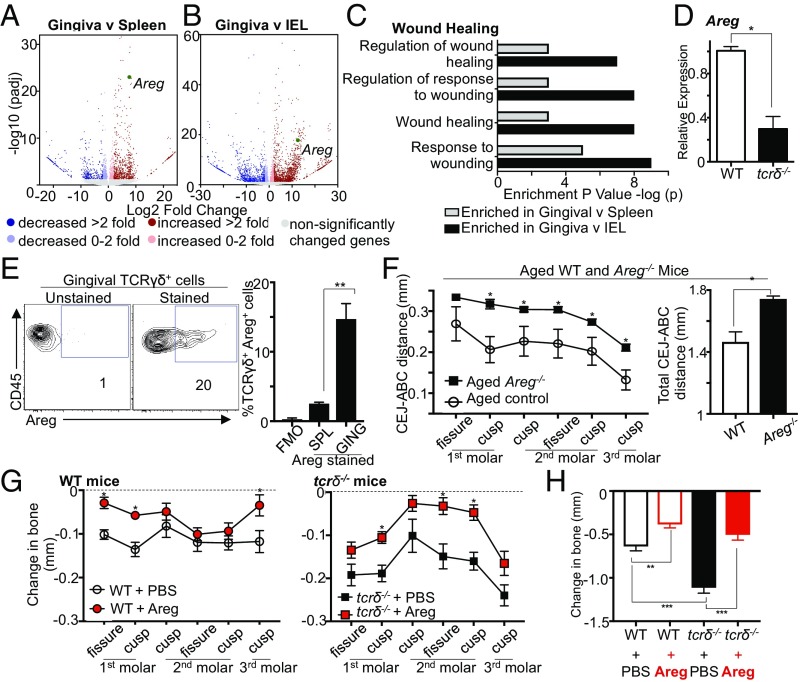
Gingival γδ T cells produce Areg to limit oral pathology. (*A* and *B*) Volcano plots comparing gene expression of gingiva versus (*A*) spleen and (*B*) intestinal epithelium (intestinal intraepithelial lymphocytes; IEL) γδ T cells. (*C*) Gene expression signatures of gingival γδ T cells were examined using PANTHER to identify enriched gene ontology terms describing biological processes. Graph outlines terms enriched in gingiva γδ T cells. (*D*) Relative expression of *Areg* in gingival tissues of wild-type and *tcrδ*^−/−^ mice. Expression in *tcrδ*^-/*−*^ gingiva presented relative to that in wild-types, data from six to seven separate mice. (*E*) Representative FACS plots gated on gingival γδ T cells stained for Areg. Cells were restimulated with PMA, ionomycin, IL-6, IL-1β, and IL-23 with Brefeldin A. (*Left*) FACS plot is unstained for Areg. (*Right*) FACS plots shows representative staining for Areg. Graph shows percentage Areg^+^TCRγδ^+^ cells in unstained (FMO), spleen (SPL), and gingiva (GING) samples from four experiments. (*F*) CEJ–ABC distances in maxilla of aged wild-type (open circles) and *Areg*^*−/−*^ mice (closed squares; *n* = 7–8 mice per group). (*Left*) CEJ–ABC distance was measured at six defined points across the molars. (*Right*) Graph shows total CEJ–ABC distance. (*G* and *H*) Experimental periodontitis was induced in control and *tcrδ*^−/−^ mice, and mice received PBS or Areg i.v. every other day three times. (*G*) CEJ–ABC distance was measured at six defined points across the molars, and changes in bone heights determined in (*Left*) control mice and (*Right*) *tcrδ*^−/−^ mice. (*H*) Graph shows total change in bone heights in ligated/periodontitis molars compared with unligated molars. Data representative of three experiments with two to five mice per group. *P < 0.05 as determined by unpaired Student’s *t* test. **P < 0.05; ***P < 0.0001, as determined by one-way ANOVA. Results are expressed as means ± SEM.

We performed genome-wide transcriptional profiling on γδ T cells flow-cytometrically purified from the gingiva, spleen (which, similar to the adult gingiva, includes Vγ1^+^ and Vγ4^+^ cells), and small intestinal epithelium (which are dominantly Vγ7^+^ cells) of naive mice. Comparison of significantly differentially expressed genes identified 841 transcripts that were altered in the gingiva compared with both small intestine and splenic γδ T cells (*SI Appendix*, Fig. S4*A*). Volcano plot visualization showed that gingival γδ T cells exhibited substantial changes in gene expression compared with those in the spleen and gut (Fig. 5 *A* and *B*). We performed biological process gene ontology on differentially expressed genes identified in pairwise comparisons to gain insight into possible gingiva-specific γδ T cell functions. This analysis revealed overrepresentation of gene sets involved in responding to microbes, with specific antimicrobial peptides and bacterial response genes elevated in gingival γδ T cells (*SI Appendix*, Fig. S4 *B* and *C*). This analysis also showed significant enrichment of gene sets associated with responses to tissue wounding (Fig. 5*C*). Indeed there was elevated expression of multiple genes shown to promote tissue repair in gingival γδ T cells (*SI Appendix*, Fig. S4*D*).

To determine the importance of these wound-healing genes in gingival homeostasis, we examined their expression in the gingiva of control and *tcrδ*^−/−^ mice. Although most of the wound healing-associated, differentially expressed genes enriched in the gingiva, as well as other genes known to be important in γδ T cell control of healing, were unchanged in the gingiva of *tcrδ*^−/−^ mice (*SI Appendix*, Fig. S4*E*), expression of *Areg* was significantly decreased in the gingiva of *tcrδ*^−/−^ mice compared with controls (Fig. 5*D*). The product of the *Areg* gene, Areg, can promote reestablishment of tissue homeostasis after injury (23–25), and its expression was significantly elevated in gingival γδ T cells (gingiva vs. spleen fold change: 7.65 padj = 9.15 × 10^−24^; gingiva vs. gut fold change: 12.54 padj = 1.63 × 10^−18^). Reduced gingival expression of *Areg* in the absence of γδ T cells implied these cells were a primary source of this wound-healing cytokine. Indeed, we found that gingival γδ T cells produced elevated levels of Areg on ex vivo stimulation compared with those from the spleen (Fig. 5*E*), and that few other gingival immune populations stained positive for Areg (*SI Appendix*, Fig. S4*F*). Interestingly most Areg^+^ gingival γδ T cells were also IL-17^+^ and CD44^+^, suggesting an IL-17^+^ γδ T cell subset made Areg in the gingiva (*SI Appendix*, Fig. S4 *G* and *H*). However, this subset was heterogeneous, using different Vγ chains, but predominately Vγ4 and Vγ6 (*SI Appendix*, Fig. S4*I*). These data identify a unique population of gingival-resident γδ T cells producing the prorepair cytokine Areg.

As Areg has been shown to support homeostasis and repair at barriers (23, 26, 27), we queried its role in promoting gingival homeostasis and limiting periodontitis. We examined periodontal pathology of aged control and *Areg*^*−/−*^ mice. In the absence of *Areg*, periodontal pathology was elevated. *Areg*^−/−^ mice exhibited increased bone loss compared with controls (Fig. 5*F*), indicating Areg is important for maintaining gingiva immune homeostasis.

Next, we determined whether Areg administration could rescue the elevated periodontal pathology seen in *tcrδ*^−/−^ mice. Acute ligature-induced periodontitis was initiated, and mice received PBS or Areg i.v. every other day. Administration of Areg during periodontitis alleviated disease pathology in both *tcrδ*^−/−^ and wild-type mice (Fig. 5*G*). Importantly, wild-type and *tcrδ*^−/−^ mice given Areg during disease now exhibited the same degree of disease pathology, with the same change in bone heights after ligature placement (Fig. 5*H*). Thus, the elevated disease pathology seen in *tcrδ*^−/−^ mice could be completely rescued by Areg administration. Combined, we demonstrate a gingiva-specific gene signature for γδ T cells highlighting Areg as a key cytokine safeguarding barrier homeostasis and limiting oral pathology.

## Discussion

Here we have identified a population of gingiva resident γδ T cells that exhibit distinct development and phenotypic profiles. Although gingiva γδ T cells are producers of the periodontitis driving cytokine IL-17, we outline an unexpected protective role for them in maintaining gingival immune homeostasis.

Our data further contribute to the idea of tissue-training of immune function, with γδ T cells in the gingiva being instructed to safeguard tissue integrity by exhibiting specific functions. This local tailoring of gingival γδ T cells could be initiated after the dramatic remodeling of the γδ T cell network after birth. The trafficking receptors supporting gingival γδ T cell recruitment during this remodeling remain unknown, yet our RNA-sequencing data show CCR1, CCR2, CCR4, CCR5, and CXCR4 are enriched on gingival γδ T cells. Nevertheless, our data do suggest that tissue damage is a key driver of this local γδ T cell remodeling. Uniquely at the gingiva, damage continually occurs as a result of mastication, which is further enhanced over the neonatal period because of tooth eruption. Indeed, gingival damage has previously been shown to instruct immune responses at this site, promoting protective Th17 responses (18). Thus, our data again outline gingival damage as an important local cue shaping homeostatic immune functions at this site.

Clearly, local commensal microbes are also a key stimulus shaping barrier immune responses. This is also the case in the gingiva (28), and our transcriptomic data suggest gingival γδ T cells respond to oral commensals. However, our data clearly indicate that changes to the oral microbial community in the absence of γδ T cells do not underscore the elevated pathology seen in *tcrδ*^−/−^ mice.

Our data demonstrate a significant enrichment of genes involved in wound healing in gingival γδ T cells. Although γδ T cells are well known to play roles in skin wound repair, our data show γδ T cells promote repair of the gingiva, a site experiencing constant damage. γδ T cell production of KGF and IGF1 support skin wound repair (14, 29). In contrast, we demonstrate a gingiva-specific gene signature for γδ T cells highlighting Areg as a key cytokine limiting oral pathology. Interestingly, most Areg^+^ γδ T cells also made IL-17, and as such, our assumption that IL-17^+^ γδ T cells would be pathogenic was incorrect. Indeed, a previous study reported protective roles for IL-17 in periodontitis (30), and many studies have shown IL-17 promotes epithelial integrity (31, 32). Nevertheless, we clearly demonstrate that γδ T cells, and specifically production of Areg, play important roles in limiting oral pathology. Indeed, we show that administration of Areg alone rescued the elevated periodontal pathology seen in *tcrδ*^−/−^ mice. A role for Areg in mucosal healing has already been suggested (23, 26, 27), but other cell types (regulatory T cells and innate lymphoid cells) were identified as Areg producers; our data show Areg production by γδ T cells, specifically those in the gingiva. Key questions remain: What are the local cues driving Areg production, and how does Areg limit periodontitis pathology and promote homeostasis? Numerous cytokines can promote T cell Areg production, including IL-33, IL-18, and PGE_2_ (23, 33, 34), all of which are expressed in the gingiva. How Areg production in the gingiva supports immune homeostasis will be more difficult to ascertain, given the many cell types Areg can stimulate and the plethora of effects this can have (25). Areg can directly support tissue repair by acting on epithelial cells and keratinocytes (35, 36). At mucosal barriers, Areg supports epithelial integrity and repair in many settings (23, 26, 27), but the molecular mechanisms driving this remain to be detailed. In addition, Areg can also enhance regulatory T cell function (37), which could also promote gingival homeostasis. Therefore, the possible targets of γδ T cell-derived Areg in the gingiva are considerable, yet our data do indicate that gingival γδ T cells themselves are not a target, as they express little EGFR.

In sum, transcriptional profiling of gingival γδ T cells demonstrated enrichment in genes contributing to wound healing. As the gingiva experiences low-level, but chronic, damage, steady-state activation of such pathways makes sense. However, we outline functions exhibited by gingival-resident γδ T cells supporting homeostasis, specifically the production of Areg, which limits development of periodontitis. Thus, we identify an important pathway regulating gingival immunity mediated by γδ T cells. Our findings highlight γδ T cells, and particularly Areg, as potential therapeutic targets to promote gingiva repair and limit periodontitis; which would have implications for both oral and systemic health.

## Materials and Methods

### Mice.

All experiments were approved by the Home Office or the Institutional Animal Care and Use Committee of the National Institute of Dental and Craniofacial Research and performed following local rules and guidelines.

### Ligature-Induced Periodontitis.

Bone loss was induced using a 5–0 silk ligature tied around the maxillary second molars, with the ligature placed in the gingival sulcus; this treatment induces bone loss, which was measured at days 8 or 10 after ligature placement.

### RNA Sequencing.

γδ T cells (1,500 cells per replicate) were FACS purified from the gingiva, small intestinal epithelium, and spleen. RNA was isolated using a single-cell RNA purification kit (Norgen Biotek) and sent to LC Sciences for sequencing.

### Statistics.

*P* values were determined with Student’s unpaired *t* test unless otherwise stated.

## Supplementary Material

Supplementary File
