# Supplementary material for: Amphiregulin-producing γδ T cells are vital for safeguarding oral barrier immune homeostasis
Source: Proc Natl Acad Sci U S A. 2018 Oct 2;115(42):10738–43. doi: 10.1073/pnas.1802320115 (PMC6196490; doi:10.1073/pnas.1802320115)
Supplement: Supplementary File [file pnas.1802320115.sapp.pdf]

Supplemental Information

SI Experimental Procedures

Mice

C57BL/6 mice were purchased from Envigo (UK) or Jackson Laboratories (USA). *Tcrδ*$^{-/-}$ and CD45.1^{+}(Pep3) mice were bred in house and *Areg*$^{-/-}$ mice were provided by Dietmar Zaiss (University of Edinburgh). Germ Free mice were obtained from the University of Manchester Gnotobiotic Facility.

Preparation of single cell suspensions

Mouse gingiva were obtained as previously described(1). In brief, gingiva was dissected and digested for 45 minutes with CollangenaseIV (Gibco) and DNase (Sigma) at 37°C with shaking. Following digestion tissue was further removed from bone pieces by scrapping using a scalpel and then mashed through a 70µm cell-strainer. Intestinal intraepithelial lymphocytes were obtained from the small intestine as previously described(2). Single cell suspensions were obtained from spleens by mashing the spleen though a 70µm cell-strainer followed by red blood cell lysis.

Flow cytometry

Single-cell preparations were stained with antibodies from eBioscience, BD Pharmingen and Biolegend. Dead cells were excluded by use of a Live/Dead fixable dye (Biolegend). Staining for Vγ chains was performed using anti-Vγ1(2.11), anti-Vγ4(UC3-10A6), and anti-Vγ5(536). Identification of Vγ6^{+} cells was performed by prestaining with GL3 antibody, followed by staining with unconjugated rabbit 17D1 IgM antibody (from David Raulet, a gift from Oliver Haworth) and a secondary anti-IgM antibody. To identify Vγ6^{+} cells, Vγ5^{+} cells were first excluded from the analysis. Samples were acquired using a Fortessa flow cytometer (BD Biosciences) and analyzed with FlowJo software (Treestar). Cell sorting was done using an Aria III (BD Biosciences).

Ex vivo re-stimulation for cytokine detection

Cells were stimulated with 50ng/ml PMA (Sigma-Aldrich) and 5µg/ml Ionomycin (Sigma-Aldrich) in the presence of GolgiPlug (Brefeldin A; BD Biosciences) for 3.5-4 hours and then stained for flow cytometric analysis. For examination of Areg production IL-1β (20ng/ml), IL-6 (50ng/ml) and IL-23 (20ng/ml) were also included during the restimulation.

www.pnas.org/cgi/doi/10.1073/pnas.1802320115

Generation of Chimeras

Bone marrow from CD45.1 mice was T cell depleted using microbeads (Miltenyi Biotec). $CD45.2^{+}$ wild-type mice were sub-lethally irradiated and reconstituted with CD45.1 bone marrow cells.

Treatment protocols during Ligature-induced Periodontitis

For some periodontitis experiments mice were treated with 7ug/mouse Areg or PBS i.v. every other day starting from the day after ligature placement. For other experiments mice were placed on antibiotics (0.3mg/ml trimethoprim and 0.7mg/ml sulfamethoxazole) in the drinking water for 5 days prior to ligature placements and maintained on this regimen throughout the experiment. For other experiments mice were treated with 200ug/treatment of anti-TCRγδ (BioXCell) every 3 days starting from 3 days before induction of periodontitis.

Bone loss Measurements

Periodontal bone heights were assessed following defleshing and staining with Methylene blue. The distance between the Cemento-enamel junction and alveolar bone crest (CEJ-ABC distance) was measured at 6 predetermined sites and combined to give a total CEJ-ABC distance presented in mm. For ligature induced periodontitis final change in bone heights was determined by subtracting the CEJ-ABC for ligated molars from un-ligated molars of mice of the same genotype.

Oral microbiome evaluation via 16S rRNA gene sequencing and qPCR

For microbiome analyses, the murine oral cavity was sampled for 30s using sterile ultra-fine swabs. Serial dilutions of the swab extracts were plated on Luria broth (aerobic growth) and Wilkins-Chalgren (anaerobic growth) agar and colony-forming units counted. Bacterial DNA from the swabs was isolated using the DNeasy Powersoil kit (Qiagen). Total 16S rRNA copy numbers were determined using described primers(3). Species-specific primers for *Aggregatibacter actinomycetemcomitans* 16S were forward GGACGGGTGAGTAATGCTTG and reverse CCTTTACCCCACCAACTACC with an annealing temperature of $58^{o}C$ as previously described(4, 5).

For 16S rRNA gene sequencing amplicon libraries were generated using previously described primers that amplify the V4 region of the 16S gene(6). Primer sequences allow a secondary nested PCR process, which incorporates Illumina adapter sequences for sequencing of samples on Illumina Sequencing platforms. Libraries were generated in triplicate before pooling. Initial processing and quality

assessment of the sequencing data was carried out at the Centre for Genomics, University of Liverpool, using an in-house pipeline. Sequences were classified using the QIIME script assign_taxonomy.py, using the RDP classifier(7) to match a representative sequence from each OTU to a sequence from the database. The pick-Rep_set.py was used to select the most abundant sequence within each OTU's cluster to use as a representative sequence. To visualize the community composition of each sample, an OTU abundance table, obtained after the normalization by rarefaction step, was used to summarize taxon abundance for each given taxonomic rank (from kingdom to species), using the QIIME script summarize_taxa.py. The OTU abundance table was also used to investigate the richness and evenness of the samples using the following estimators: total observed sequence variants (*i.e.* number of OTUs in the sample) and Shannon, Simpson, evenness. These computations were performed using the QIIME script alpha_diversity.py, considering OTUs with assigned reads only. To study how the taxa composition change in relation to groups for each metadata category (beta-diversity), the rarefied abundance table was used to build pairwise sample distance matrices, using the Bray-Curtis and the Unweighted UniFrac(8) dissimilarity measures employing the 'phyloseq' package in R(9). Differences between mouse groups were investigated using a permutational multivariate analysis of variance (PERMANOVA), performed with the 'Vegan' package in R on a Bray-Curtis distance matrix, using 1000 permutations. Significantly different OTUs between wild-type and *tcrδ*$^{-/-}$ microbiota were generated using analysis of compositions of microbiomes (ANCOM) with a false discovery rate < 0.05(10).

Real-time and conventional RT-PCR

Total RNA was obtained from gingival tissues using Trizol and cDNA synthesized using Superscript reverse transcription kit (Invitrogen/Life Technologies). To determine TCR Vγ usage, utilized primers were previously described(11). Products from PCR reactions were run on a 2% agarose gel and visualized with SYBR-safe (Invitrogen). Quantitative real-time PCR was done with SYBR green qPCR super mix (Invitrogen/Life Technologies) and normalized to *hprt* expression.

RNA-sequencing

At LC sciences, cDNAs were constructed from total RNA using the SMART-Seq v4 Ultra Low Input RNA Kit (Clontech/Takara). Libraries were prepared using Nextera XT DNA sample preparation kit (Illumina), pooled and sequenced on HiSeq X Ten

platform (Illumina). Raw fastq files were trimmed with TRIMMOMATIC(12) and aligned to the mouse GENCODE genome (GRCm38.p5) using STAR(13) (v2.5.3). Filtered reads were then counted with HTseq(14) and normalised and compared with DESeq2(15). Differential expression plots were performed with R (v3.4.1). GO analysis was performed with AmiGO2 (v2.4.26). This RNA-seq data will be accessible in the NCBI database upon publication acceptance following assignment of an accession code.

Supplemental References

1. Dutzan N, Abusleme L, Konkel JE, & Moutsopoulos NM (2016) Isolation, Characterization and Functional Examination of the Gingival Immune Cell Network. *J Vis Exp* (108):53736.
2. Konkel JE*, et al.* (2011) Control of the development of CD8alphaalpha+ intestinal intraepithelial lymphocytes by TGF-beta. *Nat Immunol* 12(4):312-319.
3. Barman M*, et al.* (2008) Enteric salmonellosis disrupts the microbial ecology of the murine gastrointestinal tract. *Infect Immun* 76(3):907-915.
4. Periasamy S & Kolenbrander PE (2009) Aggregatibacter actinomycetemcomitans builds mutualistic biofilm communities with Fusobacterium nucleatum and Veillonella species in saliva. *Infect Immun* 77(9):3542-3551.
5. Szafranski SP*, et al.* (2017) Quorum sensing of Streptococcus mutans is activated by Aggregatibacter actinomycetemcomitans and by the periodontal microbiome. *BMC Genomics* 18(1):238.
6. Caporaso JG*, et al.* (2011) Global patterns of 16S rRNA diversity at a depth of millions of sequences per sample. *Proc Natl Acad Sci U S A* 108 Suppl 1:4516-4522.
7. Wang Q, Garrity GM, Tiedje JM, & Cole JR (2007) Naive Bayesian classifier for rapid assignment of rRNA sequences into the new bacterial taxonomy. *Appl Environ Microbiol* 73(16):5261-5267.
8. Lozupone C & Knight R (2005) UniFrac: a new phylogenetic method for comparing microbial communities. *Appl Environ Microbiol* 71(12):8228-8235.
9. McMurdie PJ & Holmes S (2013) phyloseq: an R package for reproducible interactive analysis and graphics of microbiome census data. *PLoS One* 8(4):e61217.
10. Mandal S*, et al.* (2015) Analysis of composition of microbiomes: a novel method for studying microbial composition. *Microb Ecol Health Dis* 26:27663.
11. Andrew EM*, et al.* (2005) Delineation of the function of a major gamma delta T cell subset during infection. *J Immunol* 175(3):1741-1750.
12. Bolger AM, Lohse M, & Usadel B (2014) Trimmomatic: a flexible trimmer for Illumina sequence data. *Bioinformatics* 30(15):2114-2120.
13. Dobin A*, et al.* (2013) STAR: ultrafast universal RNA-seq aligner. *Bioinformatics* 29(1):15-21.
14. Edwards RA*, et al.* (2012) Real time metagenomics: using k-mers to annotate metagenomes. *Bioinformatics* 28(24):3316-3317.
15. Love MI, Huber W, & Anders S (2014) Moderated estimation of fold change and dispersion for RNA-seq data with DESeq2. *Genome Biol* 15(12):550.

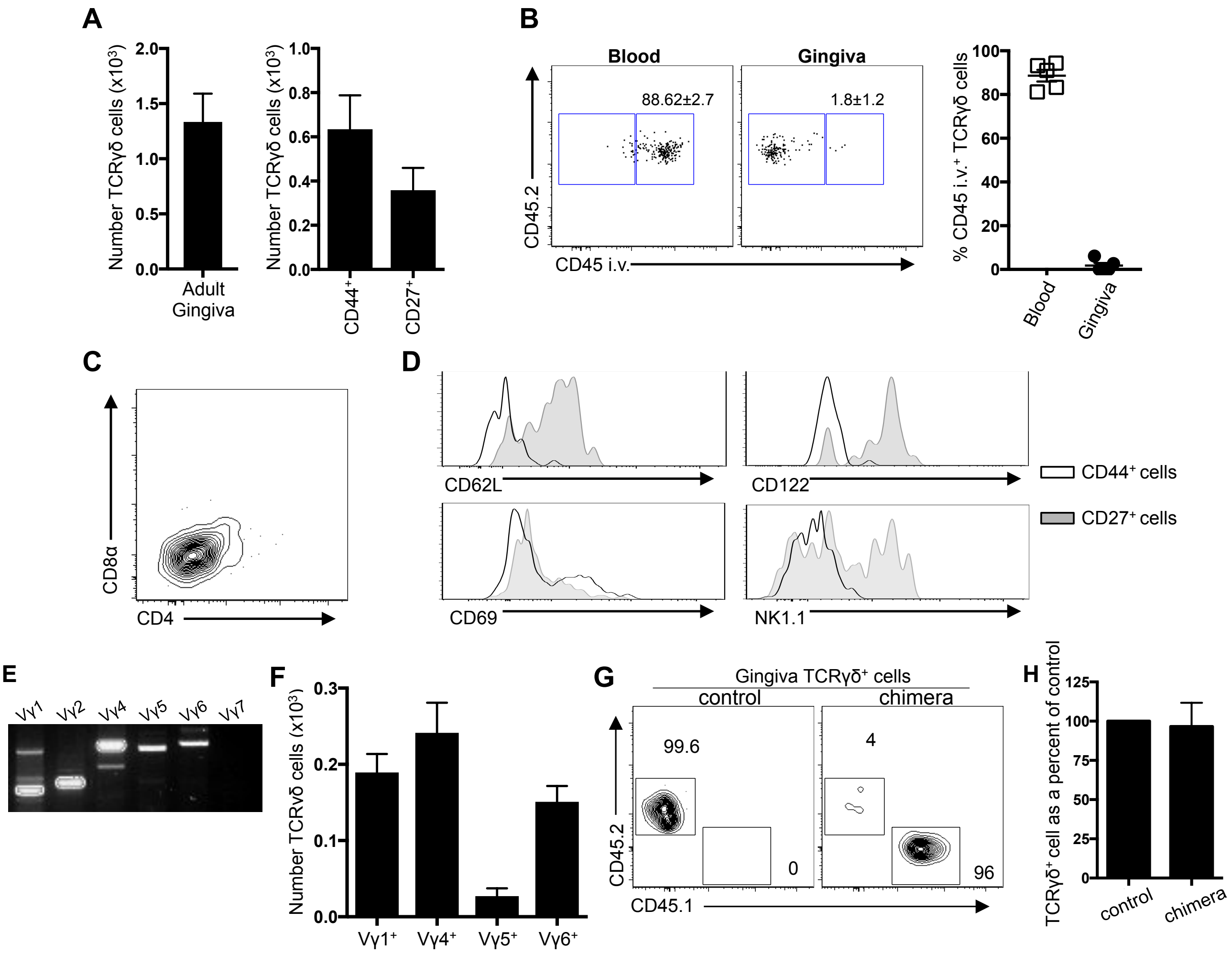

Supplemental Figure 1: CD44$^+$ and CD27$^+$ γδ T cells police the gingiva.

(**A**) Graphs show (left) total number of gingival γδ T cells in adult gingiva and (right) numbers of γδ T cells which are either CD44$^+$ or CD27$^+$ (n=6-10). (**B**) Anti-CD45 was injected i.v. into wild-type mice 5 minutes before tissue harvest. Cells were then isolated from the blood and gingiva and stained for CD45.2 and and gated on γδ T cells. FACS plots show γδ T cell staining for CD45 injected i.v. against ex vivo stained CD45.2 and bar graph shows percent of gated γδ T cells which stain positive for CD45 injected i.v. (n=5) **(C)** Representative FACS plot showing staining for CD4 and CD8α on Live, CD45$^+$, TCRγδ$^+$ gingival cells. (**D**) Representative histogram FACS plots showing staining for CD62L, CD122, CD69 and NK1.1 on CD44$^+$ (open histograms) and CD27$^+$ (grey histograms) gingival γδ T cells. (**E**) RNA from gingival tissue was analyzed for transcripts of various Vγ-chains. Gel image representative of 3 separate experiments. (**F**) Graph shows total number of specific Vγ$^+$ subsets in adult gingiva as determined by flow cytometry (n=8-11). (**G, H**) CD45.2$^+$ host mice were sub-lethally irradiated prior to receipt of CD45.1$^+$ donor bone marrow. (**G**) Representative FACS plots show degree of chimerism of γδ T cells in gingiva. (**H**) Bar graph shows the percent of γδ T cells in chimera mice relative to controls. Data representative of 2 separate experiments with 3-4 mice/group. Results are expressed as means±SEM.

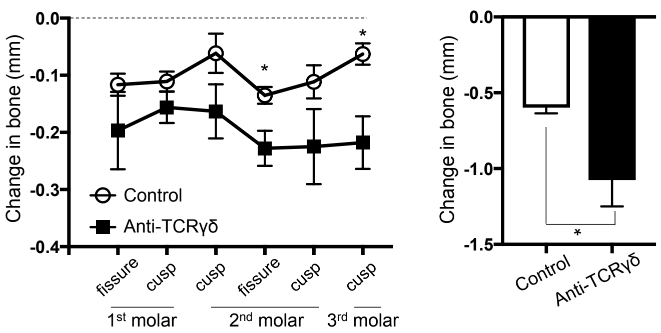

Supplemental Figure 2: Treatment with anti-TCRγδ results in exacerbated periodontitis pathology.

Experimental periodontitis was induced in mice which received 200μg anti-TCRγδ 3 days prior and 3 and 6 day post disease induction. Cemento-Enamel Junction (CEJ) to Alveolar Bone Crest (ABC) distances were measured in maxilla of control and antibody treated mice. Change in bone heights was determined by subtracting the CEJ-ABC for periodontitis/ligated molars from naïve molars of mice of the same mouse. (left) CEJ-ABC distance measured at 6 defined points across the molars. (right) Graph shows total change in bone heights in periodontitis mice compared to un-ligated controls. Data from of 2 separate experiments combined with n=3-6. *$p<0.05$ as determined by unpaired Student's *t* test. Results are expressed as means±SEM.

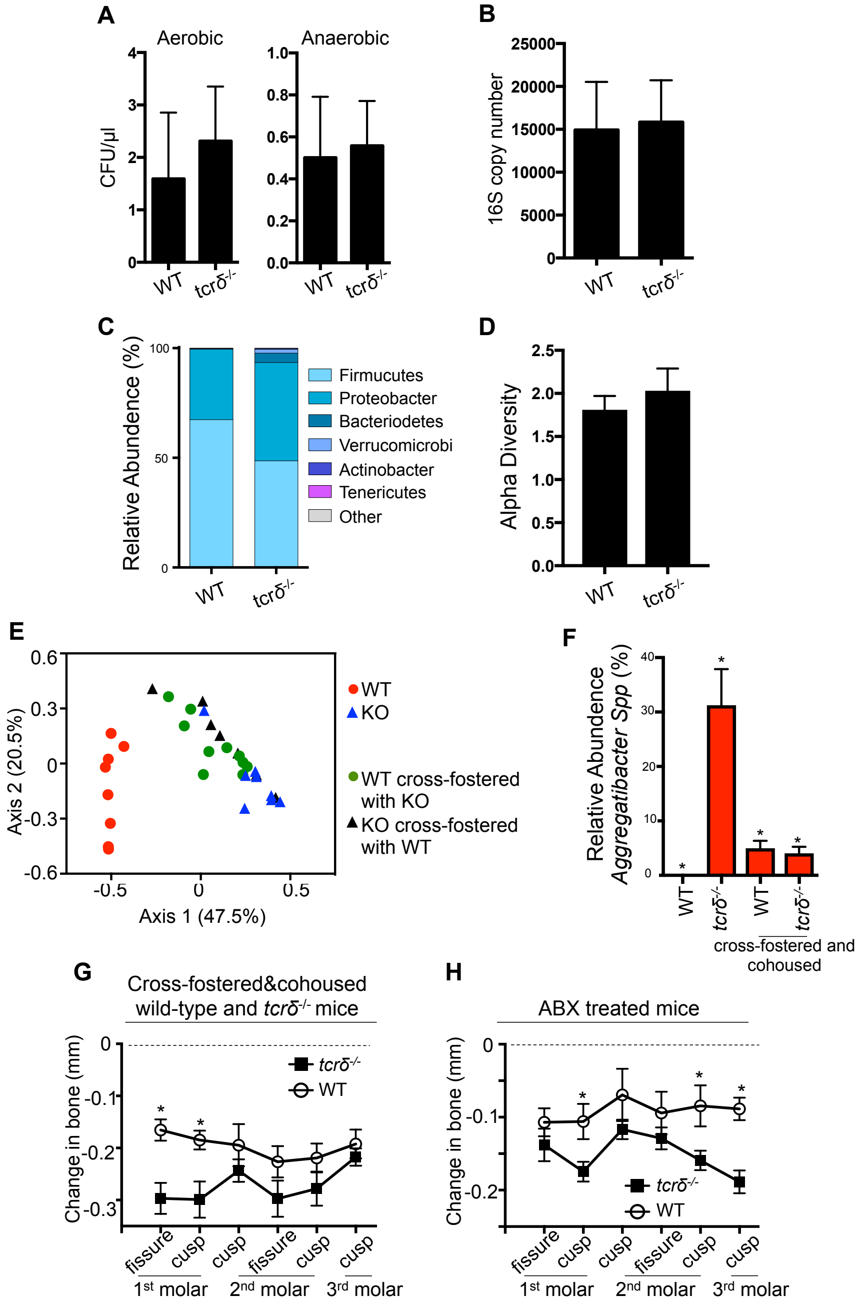
A
Aerobic
Anaerobic
CFU/µl
WT
tcrδ-/-
B
16S copy number
C
Relative Abundence (%)
Firmucutes
Proteobacter
Bacteriodetes
Verrucomicrobi
Actinobacter
Tenericutes
Other
D
Alpha Diversity
E
Axis 2 (20.5%)
Axis 1 (47.5%)
WT
KO
WT cross-fostered with KO
KO cross-fostered with WT
F
Relative Abundence Aggregatibacter Spp (%)
cross-fostered and cohoused
G
Cross-fostered&cohoused wild-type and tcrδ-/- mice
Change in bone (mm)
fissure
cusp
1st molar
2nd molar
3rd molar
H
ABX treated mice

Supplemental Figure 3: Alterations in oral microbial communities in tcr$\delta^{-/-}$ mice.

(**A**) Graphs show quantification of cultivatable oral bacteria from wild-type and *tcrδ*$^{-/-}$ mice. (**B**) Graph shows total bacterial load in the oral cavity of wild-type and *tcrδ*$^{-/-}$ mice determined by a 16S rRNA-based real-time PCR assay. **(C)** Bar graph depicts mean relative abundance of the main phyla found in the oral microbiome of wild-type control and *tcrδ*$^{-/-}$ mice. **(D)** Bar graph shows alpha diversity of oral microbial communities as determined by Shannon diversity index. (**E**) PCoA plot of the Bray-Curtis distance metric demonstrates significantly different oral microbial communities (PERMANOVA $p<0.001$) obtained from oral swab samples collected from separately housed control (red circles) and *tcrδ*$^{-/-}$ mice (blue triangles) or cross-fostered/co-housed control (green circles) and *tcrδ*$^{-/-}$ (black triangles) mice. Each symbol represents data from an individual mouse (n=7-10). **(F)** Graph showing relative abundance of OTU *Aggregatibacter spp* in separately housed and cross-fostered wild-type and *tcrδ*$^{-/-}$ mice. Differences are significant as determined by multiple comparison test after Kruskal-wallis; $p<0.001$. (**G**) Cemento-Enamel Junction (CEJ) to Alveolar Bone Crest (ABC) distances were measured in maxilla of cross-fostered/cohoused control and *tcrδ*$^{-/-}$ mice in which experimental periodontitis had been introduced. Graph shows change in bone heights across molars in periodontitis mice compared to un-ligated control. Data representative of 2 separate experiments with 3-4 mice/ group. (**H**) Experimental periodontitis was induced in control and *tcrδ*$^{-/-}$ mice that had been treated with Sulfamethoxazole-Trimethoprim for 4-5 days before induction and throughout the course of disease. CEJ to ABC distances were measured in maxilla of antibiotic treated control and *tcrδ*$^{-/-}$ mice in which experimental periodontitis had been introduced. Graph shows change in bone heights across molars in periodontitis mice compared to un-ligated controls. Data representative of 2 separate experiments with 4-6 mice/group. *$p<0.05$ as determined by unpaired Student's *t* test. Results are expressed as means±SEM.

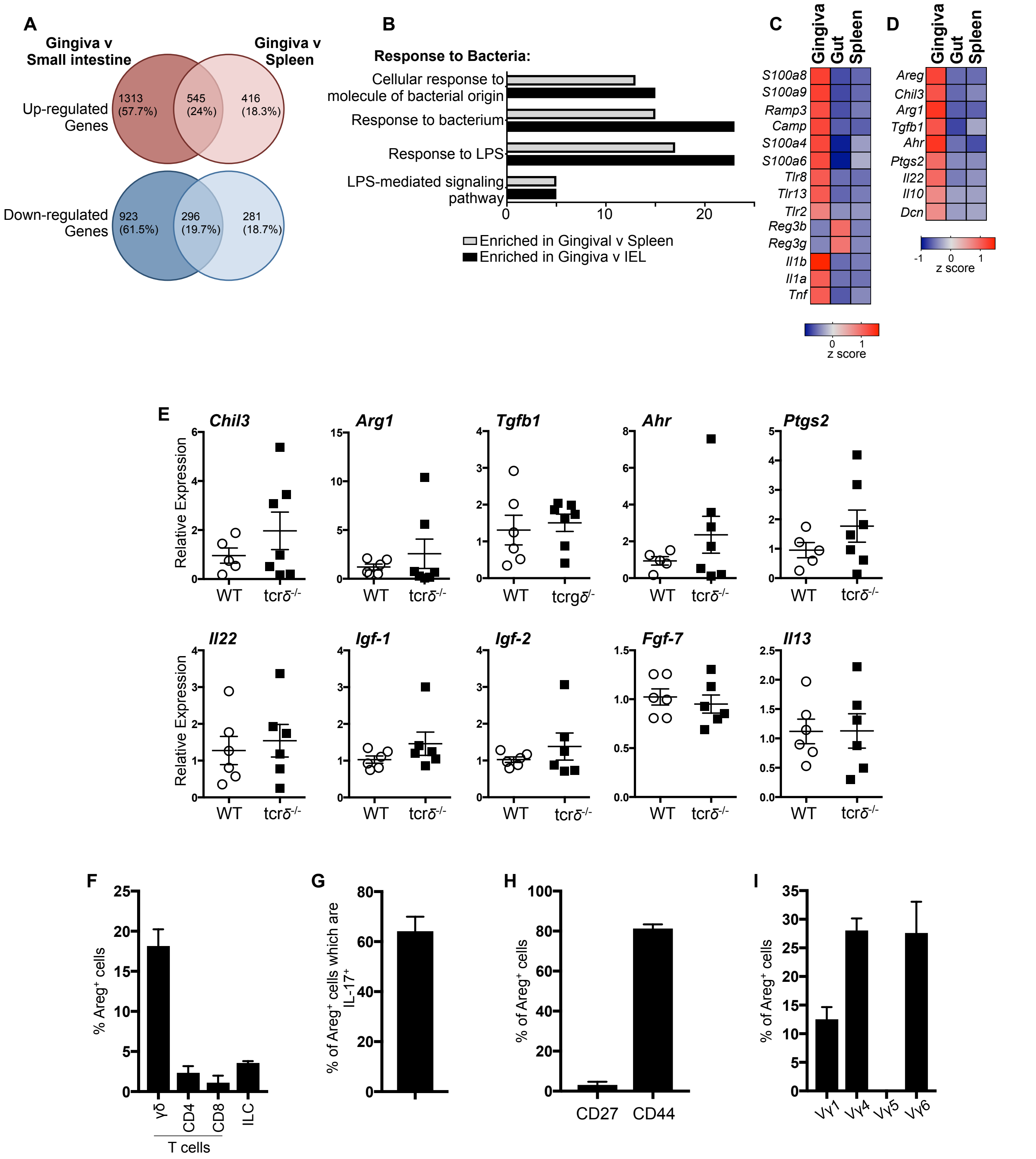
A
Gingiva v Small intestine
Gingiva v Spleen
Up-regulated Genes
1313 (57.7%)
545 (24%)
416 (18.3%)
Down-regulated Genes
923 (61.5%)
296 (19.7%)
281 (18.7%)
B
Response to Bacteria:
Cellular response to molecule of bacterial origin
Response to bacterium
Response to LPS
LPS-mediated signaling pathway
0
10
20
Enriched in Gingival v Spleen
Enriched in Gingiva v IEL
C
Gingiva
Gut
Spleen
S100a8
S100a9
Ramp3
Camp
S100a4
S100a6
Tlr8
Tlr13
Tlr2
Reg3b
Reg3g
Il1b
Il1a
Tnf
0
1
z score
D
Gingiva
Gut
Spleen
Areg
Chil3
Arg1
Tgfb1
Ahr
Ptgs2
Il22
Il10
Dcn
-1
0
1
z score
E
Chil3
Arg1
Tgfb1
Ahr
Ptgs2
Relative Expression
WT
tcrδ-/-
tcrgδ-/-
Il22
Igf-1
Igf-2
Fgf-7
Il13
Relative Expression
WT
tcrδ-/-
F
% Areg+ cells
γδ
CD4
CD8
ILC
T cells
G
% of Areg+ cells which are IL-17+
H
% of Areg+ cells
CD27
CD44
I
% of Areg+ cells
Vγ1
Vγ4
Vγ5
Vγ6

Supplemental Figure 4: Gingival γδ T cells exhibit a unique transcriptional signature.
(**A**) Venn diagram outlining number of DEG in γδ T cells sorted from the gingiva verses small intestine and the gingiva verses spleen. Blue Venn diagram indicates number of down-regulated differentially expressed genes and red Venn diagram indicates number of up-regulated differentially expressed genes. (**B**) Gene expression signatures of gingival γδ T cells were examined using PANTHER to identify enriched Gene Ontology terms describing biological processes. Graphs outline terms enriched in gingiva γδ T cells compared to spleen (Grey bars) and gut (Black bars) γδ T cells. (**C,D**) Heat maps representing z-score of indicated genes associated with (**C**) responses to bacteria and (**D**) responses to wounding. RNA sequencing data is representative of sequencing of three sets of γδ T cells FACS purified from each indicated tissue. (**E**) Relative expression of indicated genes in gingival tissues of wild-type and *tcrδ*$^{-/-}$ mice. Expression in *tcrδ*$^{-/-}$ gingiva presented relative to that in wild-type control (each dot represents one mouse). (**F-I**) Bar graph shows percent of gingival cells staining positive for Areg following *ex vivo* restimulation with PMA, ionomycin, IL-6, IL-1β, and IL-23 in the presence of Brefeldin A (n=4-10). (**F**) Bar graph shows the percent of different lymphocyte populations staining positive for Areg. (**G**) Bar graph shows the percent of Areg^{+} γδ T cells positive for IL-17. (**H**) Bar graph shows the percent of Areg^{+} γδ T cells positive for CD27 and CD44. (**I**) Bar graph shows the frequencies of specific Vγ^{+} subsets in gated Areg^{+} γδ T cells. Results are expressed as means±SEM.

Group	OTU	Taxonomy
OTU significantly enriched in $tcr\delta^{-/-}$ mice	OTU2278	k__Bacteria; p__Proteobacteria; c__Gammaproteobacteria; o__Pasteurellales; f__Pasteurellaceae; g__Aggregatibacter
	OTU1632	k__Bacteria; p__Proteobacteria; c__Gammaproteobacteria; o__Pasteurellales; f__Pasteurellaceae; g__Aggregatibacter
	OTU2532	k__Bacteria; p__Proteobacteria; c__Gammaproteobacteria; o__Pasteurellales; f__Pasteurellaceae; g__Aggregatibacter
	OTU2117	k__Bacteria; p__Firmicutes; c__Bacilli; o__Lactobacillales; f__Streptococcaceae; g__Streptococcus
	OTU3109	k__Bacteria; p__Proteobacteria; c__Gammaproteobacteria; o__Pasteurellales; f__Pasteurellaceae; g__Aggregatibacter
	OTU3142	k__Bacteria; p__Firmicutes; c__Bacilli; o__Lactobacillales; f__Streptococcaceae; g__Streptococcus; s__
	OTU2658	k__Bacteria; p__Proteobacteria; c__Gammaproteobacteria; o__Pasteurellales; f__Pasteurellaceae; g__Aggregatibacter
	OTU281	k__Bacteria; p__Firmicutes; c__Bacilli; o__Lactobacillales; f__Streptococcaceae; g__Streptococcus; s__
	OTU510	k__Bacteria; p__Proteobacteria; c__Gammaproteobacteria; o__Pasteurellales; f__Pasteurellaceae; g__Aggregatibacter
	OTU1952	k__Bacteria; p__Firmicutes; c__Bacilli; o__Lactobacillales; f__Streptococcaceae; g__Streptococcus; s__
	OTU3086	k__Bacteria; p__Proteobacteria; c__Gammaproteobacteria; o__Pasteurellales; f__Pasteurellaceae; g__Aggregatibacter
	OTU2311	k__Bacteria; p__Firmicutes; c__Bacilli; o__Lactobacillales; f__Streptococcaceae; g__Streptococcus
	OTU1890	k__Bacteria; p__Proteobacteria; c__Gammaproteobacteria; o__Pasteurellales; f__Pasteurellaceae; g__Aggregatibacter
	OTU2910	k__Bacteria; p__Proteobacteria; c__Gammaproteobacteria; o__Pasteurellales; f__Pasteurellaceae; g__Aggregatibacter
	OTU662	k__Bacteria; p__Firmicutes; c__Bacilli; o__Lactobacillales; f__Streptococcaceae; g__Streptococcus
	OTU1198	k__Bacteria; p__Proteobacteria; c__Gammaproteobacteria; o__Pasteurellales; f__Pasteurellaceae; g__Aggregatibacter
	OTU3356	k__Bacteria; p__Proteobacteria; c__Gammaproteobacteria; o__Pasteurellales; f__Pasteurellaceae
	OTU745	k__Bacteria; p__Firmicutes; c__Bacilli; o__Lactobacillales; f__Streptococcaceae; g__Streptococcus
	OTU1305	k__Bacteria; p__Proteobacteria; c__Gammaproteobacteria; o__Pasteurellales; f__Pasteurellaceae; g__Aggregatibacter
	OTU2745	k__Bacteria; p__Proteobacteria; c__Gammaproteobacteria; o__Pasteurellales; f__Pasteurellaceae; g__Aggregatibacter
	OTU2113	k__Bacteria; p__Proteobacteria; c__Gammaproteobacteria; o__Pasteurellales; f__Pasteurellaceae; g__Aggregatibacter
	OTU2523	k__Bacteria; p__Proteobacteria; c__Gammaproteobacteria; o__Pasteurellales; f__Pasteurellaceae; g__Aggregatibacter
	OTU1133	k__Bacteria; p__Proteobacteria; c__Gammaproteobacteria; o__Pasteurellales; f__Pasteurellaceae; g__Aggregatibacter
	OTU2239	k__Bacteria; p__Proteobacteria; c__Gammaproteobacteria; o__Pasteurellales; f__Pasteurellaceae; g__Aggregatibacter
	OTU436	k__Bacteria; p__Firmicutes; c__Bacilli; o__Lactobacillales; f__Streptococcaceae; g__Streptococcus
	OTU3059	k__Bacteria; p__Proteobacteria; c__Gammaproteobacteria; o__Pasteurellales; f__Pasteurellaceae; g__Aggregatibacter
	OTU907	k__Bacteria; p__Firmicutes; c__Bacilli; o__Lactobacillales; f__Streptococcaceae; g__Streptococcus; s__
	OTU1096	k__Bacteria; p__Firmicutes; c__Bacilli; o__Lactobacillales; f__Streptococcaceae; g__Streptococcus; s__
	OTU1990	k__Bacteria; p__Proteobacteria; c__Gammaproteobacteria; o__Pasteurellales; f__Pasteurellaceae; g__Aggregatibacter
	OTU3122	k__Bacteria; p__Firmicutes; c__Bacilli; o__Lactobacillales; f__Streptococcaceae; g__Streptococcus; s__
	OTU1106	k__Bacteria; p__Firmicutes; c__Bacilli; o__Lactobacillales; f__Streptococcaceae; g__Streptococcus; s__
	OTU3181	k__Bacteria; p__Firmicutes; c__Bacilli; o__Lactobacillales; f__Streptococcaceae; g__Streptococcus; s__
	OTU1112	k__Bacteria; p__Firmicutes; c__Bacilli; o__Lactobacillales; f__Streptococcaceae; g__Streptococcus; s__
	OTU1223	k__Bacteria; p__Proteobacteria; c__Gammaproteobacteria; o__Pasteurellales; f__Pasteurellaceae
	OTU3421	k__Bacteria; p__Firmicutes; c__Bacilli; o__Lactobacillales; f__Streptococcaceae; g__Streptococcus; s__
	OTU537	k__Bacteria; p__Proteobacteria; c__Gammaproteobacteria; o__Pasteurellales; f__Pasteurellaceae; g__Aggregatibacter
	OTU2467	k__Bacteria; p__Proteobacteria; c__Gammaproteobacteria; o__Pasteurellales; f__Pasteurellaceae; g__Aggregatibacter
	OTU1857	k__Bacteria; p__Firmicutes; c__Bacilli; o__Lactobacillales; f__Streptococcaceae; g__Streptococcus; s__
	OTU2152	k__Bacteria; p__Firmicutes; c__Bacilli; o__Lactobacillales; f__Streptococcaceae; g__Streptococcus; s__
	OTU2220	k__Bacteria; p__Firmicutes; c__Bacilli; o__Lactobacillales; f__Streptococcaceae; g__Streptococcus; s__
	OTU2612	k__Bacteria; p__Firmicutes; c__Bacilli; o__Lactobacillales; f__Streptococcaceae; g__Streptococcus; s__
	OTU2699	k__Bacteria; p__Firmicutes; c__Bacilli; o__Lactobacillales; f__Streptococcaceae; g__Streptococcus; s__
	OTU1573	k__Bacteria; p__Firmicutes; c__Bacilli; o__Lactobacillales; f__Streptococcaceae; g__Streptococcus
	OTU496	k__Bacteria; p__Firmicutes; c__Bacilli; o__Lactobacillales; f__Streptococcaceae; g__Streptococcus; s__
	OTU346	k__Bacteria; p__Firmicutes; c__Bacilli; o__Lactobacillales; f__Streptococcaceae; g__Streptococcus; s__
	OTU2281	k__Bacteria; p__Proteobacteria; c__Gammaproteobacteria; o__Pasteurellales; f__Pasteurellaceae
	OTU3229	k__Bacteria; p__Firmicutes; c__Bacilli; o__Lactobacillales; f__Streptococcaceae; g__Streptococcus; s__
	OTU3612	k__Bacteria; p__Firmicutes; c__Bacilli; o__Lactobacillales; f__Streptococcaceae; g__Streptococcus; s__
OTU significantly enriched in wild-type mice	OTU2173	k__Bacteria; p__Proteobacteria; c__Gammaproteobacteria; o__Pasteurellales; f__Pasteurellaceae
	OTU570	k__Bacteria; p__Firmicutes; c__Bacilli; o__Lactobacillales; f__Lactobacillaceae; g__Lactobacillus
	OTU499	k__Bacteria; p__Proteobacteria; c__Gammaproteobacteria; o__Oceanospirillales; f__Halomonadaceae; g__Halomonas
	OTU2298	k__Bacteria; p__Firmicutes; c__Bacilli; o__Lactobacillales; f__Lactobacillaceae; g__Lactobacillus
	OTU832	k__Bacteria; p__Firmicutes; c__Bacilli; o__Lactobacillales; f__Streptococcaceae; g__Streptococcus
	OTU1173	k__Bacteria; p__Firmicutes; c__Bacilli; o__Lactobacillales; f__Lactobacillaceae; g__Lactobacillus
	OTU2538	k__Bacteria; p__Proteobacteria; c__Gammaproteobacteria; o__Pasteurellales; f__Pasteurellaceae
	OTU2550	k__Bacteria; p__Firmicutes; c__Bacilli; o__Lactobacillales; f__Streptococcaceae; g__Streptococcus; s__
	OTU2120	k__Bacteria; p__Firmicutes; c__Bacilli; o__Lactobacillales; f__Streptococcaceae; g__Streptococcus
	OTU1120	k__Bacteria; p__Firmicutes; c__Bacilli; o__Lactobacillales; f__Streptococcaceae; g__Streptococcus
	OTU1753	k__Bacteria; p__Proteobacteria; c__Gammaproteobacteria; o__Pasteurellales; f__Pasteurellaceae
	OTU2102	k__Bacteria; p__Firmicutes; c__Bacilli; o__Lactobacillales; f__Streptococcaceae; g__Streptococcus; s__
	OTU3381	k__Bacteria; p__Firmicutes; c__Bacilli; o__Lactobacillales; f__Streptococcaceae; g__Streptococcus; s__
	OTU3692	k__Bacteria; p__Firmicutes; c__Bacilli; o__Lactobacillales; f__Streptococcaceae; g__Streptococcus; s__
	OTU692	k__Bacteria; p__Firmicutes; c__Bacilli; o__Lactobacillales; f__Streptococcaceae; g__Streptococcus; s__
	OTU1712	k__Bacteria; p__Firmicutes; c__Bacilli; o__Lactobacillales; f__Streptococcaceae; g__Streptococcus
	OTU2662	k__Bacteria; p__Firmicutes; c__Bacilli; o__Lactobacillales; f__Streptococcaceae; g__Streptococcus
	OTU1292	k__Bacteria; p__Firmicutes; c__Bacilli; o__Lactobacillales; f__Streptococcaceae; g__Streptococcus; s__
	OTU1524	k__Bacteria; p__Firmicutes; c__Bacilli; o__Lactobacillales; f__Streptococcaceae; g__Streptococcus; s__
	OTU571	k__Bacteria; p__Firmicutes; c__Bacilli; o__Lactobacillales; f__Streptococcaceae; g__Streptococcus; s__
	OTU1383	k__Bacteria; p__Firmicutes; c__Bacilli; o__Lactobacillales; f__Streptococcaceae; g__Streptococcus; s__
	OTU119	k__Bacteria; p__Firmicutes; c__Bacilli; o__Lactobacillales; f__Streptococcaceae; g__Streptococcus; s__
	OTU1500	k__Bacteria; p__Firmicutes; c__Bacilli; o__Lactobacillales; f__Streptococcaceae; g__Streptococcus; s__
	OTU1088	k__Bacteria; p__Firmicutes; c__Bacilli; o__Lactobacillales; f__Streptococcaceae; g__Streptococcus; s__
	OTU438	k__Bacteria; p__Firmicutes; c__Bacilli; o__Lactobacillales; f__Streptococcaceae; g__Streptococcus; s__
	OTU2070	k__Bacteria; p__Firmicutes; c__Bacilli; o__Lactobacillales; f__Streptococcaceae; g__Streptococcus
	OTU3668	k__Bacteria; p__Firmicutes; c__Bacilli; o__Lactobacillales; f__Streptococcaceae; g__Streptococcus; s__
	OTU3080	k__Bacteria; p__Firmicutes; c__Bacilli; o__Lactobacillales; f__Streptococcaceae; g__Streptococcus

Table 1: Alterations in oral microbial communities in tcr*δ*$^{-/-}$ mice.
Table shows significantly enriched OTU as determined by a pairwise comparison of raw reads from oral microbial communities of separately housed wild-type control and *tcrδ*$^{-/-}$ mice. Blue bar indicates OTU significantly enriched in *tcrδ*$^{-/-}$ mice and red bar indicates OTU significantly enriched in wild-type mice as determined by ANCOM analysis with a false discovery rate <0.05.
